# Salivary cortisol as a biomarker of stress in surgical patients

**DOI:** 10.5937/jomb0-42011

**Published:** 2023-08-25

**Authors:** Sanja Vicković, Ranko Zdravković, Sanja Maričić-Prijić, Dragan Nikolić, Dragana Pap, Emina Čolak, Snežana Jovičić

**Affiliations:** 1 University Clinical Center of Vojvodina, Novi Sad; 2 University of Novi Sad, Faculty of Medicine, Novi Sad; 3 Institute of Cardiovascular Diseases of Vojvodina, Sremska Kamenica; 4 Students Health Protection Institute, Faculty of Pharmacy, Novi Sad; 5 University Clinical Center of Serbia, Institute of Medical Biochemistry, Belgrade; 6 University of Belgrade, Faculty of Pharmacy Belgrade, Belgrade

**Keywords:** postoperative pain, salivary cortisol, serum cortisol, surgical stress, postoperativni bol, salivarni kortizol, serumski kortizol, hirurški stres

## Abstract

**Background:**

Surgical stress and pain result in activation of hypothalamus-pituitary-adrenal axis. The aim of this study was to establish the effects of postoperative pain and various modalities of analgesic administration on salivary and serum cortisol levels, as well as to establish the validity of salivary cortisol as a stress indicator in surgical patients.

**Methods:**

A randomized controlled trial involved 60 patients scheduled for elective abdominal aortic aneurysm surgery. Patients were randomly divided into two groups depending on the model of postoperative analgesia. The first group (MI - morphine intermittently) included patients given morphine doses 0.1 mg/kg/6h s.c. intermittently. The second group (MPCA - morphine patient-controlled analgesia) included patients who received morphine via the PCA system - intravenous administration of morphine adjusted to a dose of 1 mg per shot and a lockout interval of 6 minutes.

## Abstract (sr)

**Uvod:**

Hirurški stres i bol su uzrok aktivacije hipotalamusno-hipofizno-nadbubrežne osovine. Cilj ove studije bio je da se utvrdi uticaj postoperativnog bola i različitih vidova administracije analgetika na vrednosti kortizola u serumu i salivi, kao i da se utvrdi da li je salivarni kortizol dobar pokazatelj stresa kod hirurških pacijenata.

**Metode:**

Randomizovana kontrolisana studija koja je uključila 60 pacijenata primljenih za elektivnu operaciju aneurizme abdominalne aorte. Pacijenti su metodom slučajnog izbora podeljeni u dve grupe u zavisnosti od modela postoperativne analgezije. Prvu grupu (MI - intermitentna primena morfina) su činili pacijenti koji su dobijali morfin intermitentno u dozi od 0.1 mg/kg/6h s.c. U drugoj grupi (MPCA - analgezija morfinom kontrolisana od strane pacijenta) su bili pacijenti koji su dobijali morfin takozvanom PCA metodom - intravenska primena morfina 1 pritisak/1 mg, interval 6 minuta.

**Rezultati:**

Intenzitet bola se nije značajno razlikovao do desetog sata nakon operacije. Međutim, u periodu od desetog do osamnaestog sata nakon operacije bol je bio izraženiji u MPCA grupi (P < 0.05). Hemodinamska nestabilnost je bila zastupljenija u grupi MI (40.0% vs 6.7%, P = 0.0048). Serumski kortizol bio je gotovo identičan po grupama (MI 509.4 nmol/L vs MPCA 511.0 nmol/L, P = 0.1473). Salivarni kortizol je bio viši u grupi MPCA ali lika nije statistički značajna (47.1 nmol/L vs 116.3 nmol/L, P = 0.0970).

**Zaključak:**

Naša studija je pokazala da je salivarni kortizol senzitivniji biomarker stresa kod hirurških pacijenata u odnosu na serumski kortizol.

## Introduction

Abdominal aortic aneurysm (AAA) is a vascular pathology characterized by dilatation of the aorta with a diameter of >3 cm or 50% greater than the normal diameter, which poses a risk of aortic rupture. Ultrasound screening among the population over 65 revealed that 4–7% of males and 1–2% females suffer from AAA [1]. The majority of aneurisms are asymptomatic until the rupture, when it becomes life-threatening condition with a mortality rate of even 50% [2]. Surgical management is indicated when the aneurysm diameter is 5.5 cm or larger in men and 5.0 cm or larger in women [3]. Open surgical repair and endovascular placement of a stent graft are currently considered standard approach to AAA management [4].

The surgery causes postoperative pain, which should be managed as soon as possible and with a highest possible efficacy in order to reduce suffering, provide adequate recovery and prevent complications [5]. Despite considerable achievements in the therapy of acute postoperative pain during past few decades, a non-negligible percentage of patients are still reporting a moderate to severe postoperative pain [6]. Having in mind that the most severe pain intensity is present immediately after surgery, the process of healing could hardly be imagined without opioid analgesics. Morphine still remains a most frequently used opioid for postoperative analgesia compared to lipophilic opioids (fentanyl, remifentanil, alfentanil and sufentanil), which have a faster onset but also a shorter duration of action [6]. Analgesic therapy can be conducted by intermittent administration at certain time intervals or by PCA (patient-controlled analgesia) technique. PCA is a frequently used method of analgesia in which the patient administers predetermined doses to himself. PCA can minimize the occurrence of gaps in analgesic administration, providing more uniform analgesia and eliminating painful waiting periods between requesting and receiving the medication [7].

Surgical stress and pain result in activation of hypothalamus-pituitary-adrenal (HPA) axis [8]. Stimulation of HPA axis leads to a production of corticotrophin-releasing factor (CRF) in the hypothalamus, which in turn stimulates adrenocorticotropic secretion by the hypophysis and leads to a consequent stimulation of adrenal gland cortex to release cortisol, one of the major glucocorticoids [9] [10]. Cortisol concentration can be measured in blood serum, urine, cerebrospinal fluid, sweat and saliva [11]. Salivary cortisol is nowadays used as a biomarker of psychological stress and some mental and physical diseases. It has also been used to research basal cortisol patterns in neonates and in evaluating their response to stress [12]. However, salivary cortisol levels are partially dissociated from CRF, adrenocorticotropic hormone, and serum cortisol levels [13].

The aim of this study was to investigate and compare the efficacy and safety of PCA compared to intermittent administration of bolus doses of morphine after AAA repair surgery, to establish the effects of postoperative pain and various modalities of analgesic administration on salivary and serum cortisol levels, as well as to establish the validity of salivary cortisol as a stress indicator or predictive biomarker in surgical patients.

## Materials and methods

The study protocol complied with the Declaration of Helsinki. After obtaining approval from the Institutional Ethics Commission and informed written patient consent, a total of 60 patients of both sexes scheduled for elective AAA repair were included in this prospective study. The study was conducted at the Clinic for Anesthesia, Intensive Therapy and Pain Management of the University Clinical Center of Vojvodina. The patients with Cushing’s syndrome and patients on corticosteroid therapy were not included in the study. Patients on prolonged mechanical ventilation as well as those who were non-cooperative were excluded from the study.

Depending on the postoperative analgesia model the patients were distributed into two groups by the method of random selection. The first group (MI) included patients given morphine doses 0.1 mg/kg/6h s.c. intermittently. The second group (MPCA) included patients who received morphine via the PCA system – intravenous administration of morphine adjusted to a dose of 1 mg per shot and a lockout interval of 6 minutes. In case of need for additional analgesia, the patients received metamizole sodium and acetaminophen.

### Anesthesia and surgical technique

All patients were operated under general balanced anesthesia. Anesthetic premedication was done using midazolam 0.05 mg/kg i.m. Propofol 2 mg/kg and rocuronium bromide 0.6 mg/kg were used for induction to anesthesia. The anesthesia and analgesia were maintained applying sevoflurane and fentanyl, respectively. Muscle relaxation was provided by continuous infusion of rocuronium bromide 0.4 mg/kg.

The operations were performed by open surgical repair, with a transperitoneal approach through a midline laparotomy. Repair of the aorta was carried out in a systematic fashion that involves clamping the aorta to stop blood flow, the aneurysmal segment is removed and suturing a synthetic graft to replace the diseased arterial segment.

### Pain assessment

The intensity of pain has been assessed using Numerical rating scale (NRS) at one-hour intervals starting from the hour 3 post-surgery during first 12 hours, and then at hours 15 and 18 post surgery. NRS values were defined on an eleven-point scale with a range 0–10 (0 = no pain, 10 = worst pain). The patient was asked to encircle the number corresponding to his/her pain intensity. Score 1–3 was interpreted as mild pain, score 4–6 as moderate, and score 7–10 as severe pain.

### Assessment of patient satisfaction with postoperative analgesia

The patient satisfaction with postoperative analgesia was assessed 24 hours after surgery by asking the patients to evaluate the quality of postoperative analgesia using the grading scale 1 to 10.

### Demographic, clinical, and laboratory measures

The following data were registered: age, sex, and comorbidities. One day post-surgery, in all patients, salivary cortisol and serum cortisol levels were measured at 6:00 am and 8:00 am, respectively. Salivary cortisol was sampled before serum cortisol to avoid the influence of venipuncture stress on salivary cortisol values. Abstaining from any food, drink, chewing gum, tooth brushing or using dental floss for minimum of 60 minutes before sampling is the prerequisite for a successful collection of saliva samples. The saliva samples were collected into salivettes (Salivette®) according to the prescribed procedure as following: The top cap of the tube was removed to expose the swab. The swab was introduced into the mouth and rolled in the mouth (left, right, forward) for approximately two minutes. After two minutes, the tampon was spitted back into the tube; the tube cap was put back and screwed on tightly and immediately sent to the laboratory for testing. Serum cortisol was determined applying CMIA (Chemiluminescent Micro particle Immunoassay) method, whereas ECLIA (Electro-Chemiluminescence Immunoassay) was used for determining salivary cortisol. Both techniques are innovative serological methods of new generation, fully automated, showing superior specificity and sensitivity than the ELISA. These methods are effective tools for determining supernatant or serum levels of hormones, tumor markers and other biomarkers.

### Statistical analyses

Statistical analysis was performed applying the R software (Version 3.4.0). The following descriptive statistic measures were used: arithmetic mean, standard deviation, frequency and percentages. A t-test for independent samples and a Mann-Whitney test were used to compare the mean values of the variables of the two populations. Correlation was determined using Spearman’s correlation coefficient. A p < 0.05 value was taken for statistical significance of the test.

## Results

Both groups consisted of 30 patients. The group MI included 27 male and 3 female patients, whereas MPCA group consisted of 26 males and 4 females. The average age in group MI was 68.1 and 64.5 in MPCA group. The average weight and height of MI-patients was 82.6 kg and 172.3 cm, and 85.3 kg and 176.1 cm in group MPCA. The groups did not differ with respect to the identified comorbidities (Table 1).

**Table 1:** Demographic, anthropometric characteristics and comorbidities. There were no statistically significant differences between the groups.

Variable	MI	MPCA
Patients n	30	30
Male n (%)<br>Female n (%)	27 (90)<br>3 (10)	26 (86.7)<br>4 (13.3)
Age (years), mean (SD)	68.1 (7.6)	64.5 (6.6)
Weight (kg), mean (SD)	82.6 (7.0)	85.3 (9.8)
Height (cm), mean (SD)	172.3 (6.2)	176.1 (7.9)
Hypertension n (%)	25 (83.3)	26 (86.7)
Diabetes mellitus n (%)	5 (16.7)	7 (23.3)
History of myocardial<br>infarction n (%)	4 (13.3)	5 (16.7)
History of cerebrovascular<br>accident n (%)	1 (3.3)	1 (3.3)
Hyperlipoproteinemia n (%)	6 (20)	4 (13.3)
Chronic obstructive<br>pulmonary disease n (%)	2 (6.7)	4 (13.3)
Hypothyroidism n (%)	2 (6.7)	0 (0)

**Figure 1:**
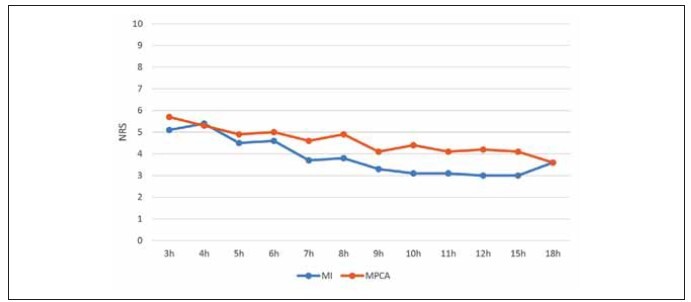
Average intensity of pain (numerical rating scale 1–10) per hour after surgery in the MI and MPCA groups.

The average NRS pain intensity according to groups is displayed in Figure 1. The intensity of pain mostly ranged between 3 and 6, which is considered mild to moderate. It did not significantly vary until the hour 10 post-surgery. However, in the period from hour 10 to hour 18 post-surgery, higher pain intensity was reported in group MPCA (P < 0.05). In hour 18 post-surgery, the reported pain intensity equalized in both groups. According to the data shown in Table 2, the higher percentage of MI-patients required additional analgesics as compared with the MPCA group. Even 64.3% of patients from group MI required full dose of analgesics (metamizole sodium 5 g/acetaminophen 3 g), whereas the same dosage was required by 46.2% of MPCA-patients.

**Table 2:** Addition of analgesics by groups.

Analgesic	MI	MPCA
Metamizol natrium 5 g	2 (7.1%)	4 (15.4%)
Metamizol natrium 5 g<br>Acetaminophen 2 g	2 (7.1%)	0 (0%)
Metamizol natrium 5 g<br>Acetaminophen 3 g	18 (64.3%)	12 (46.2%)
Acetaminophen 2 g	2 (7.1%)	0 (0%)
Acetaminophen 3 g	4 (14.3%)	10 (38.5)
**Total**	**28 (100%)**	**26 (100%)**

Hemodynamic instability is defined as a deviation greater than 30% in relation to the basal values of blood pressure and heart rate, and it was more prevalent in the MI group (40.0% vs 6.7%, P = 0.0048).

Patients’ satisfaction after the investigation period was equal (MI 8.3 vs MPCA 8.2, P = 0.8361). None of the patients experienced side effects of morphine analgesia, and we believe that morphine administered by any of these methods is absolutely safe after abdominal aortic aneurysm surgery.

**Figure 2:**
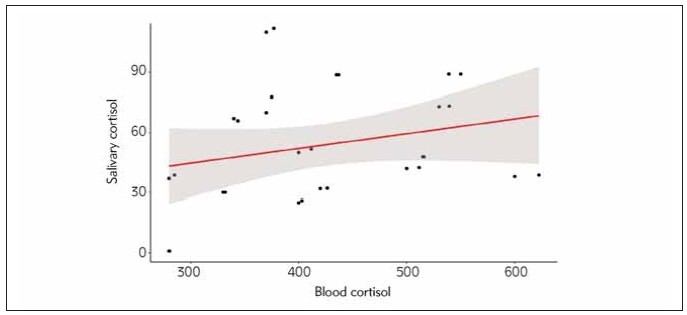
Spearman’s Rank correlation coefficient between blood and salivary cortisol in examined patients. The correlation is positive and marginally significant (P = 0.05781).

Serum cortisol levels were almost identical in both groups (MI 509.4 vs MPCA 511.0 nmol/L, P = 0.1473) (Table 3). Higher values of salivary cortisol were recorded in group MPCA; however, the difference was not statistically significant (47.1 vs 116.3 nmol/L, P = 0.0970). Spearman’s Rank correlation coefficient between the serum cortisol and salivary cortisol revealed positive correlation (0.35) on the borderline of statistical significance (P = 0.05781) (Figure 2).

**Table 3:** Cortisol values in blood and saliva by groups.

Variable	Normal<br>range	MI	MPCA	P value
Blood cortisol at<br>8 a.m. nmol/L,<br>mean (SD)	101.2–535.7	509.4<br>(49.6)	511.0<br>(128.4)	0.1473
Salivary cortisol<br>at 6 a.m.<br>nmol/L,<br>mean (SD)	<20.3	47.1<br>(27.8)	116.3<br>(151.8)	0.0970

## Discussion

In spite of most recent trend of using non-opioid anesthesia and analgesia as well as oral and regional analgesia, opioids are still the most commonly used analgesics for the management of post-operative pain. When opioids are indicated, PCA is the method of choice [14]. The well-established benefits of PCA techniques are dose adaptation to individual needs by giving the patients control over their own pain, relatively fast onset of action, predictable drug delivery, and high scores of satisfaction [6]. A large-scale study conducted in hospitals in Germany revealed that this technique is used as the method of choice at 79% of clinics [15]. The results of 49 studies indicated the superiority of PCA over opioid non-PCA in view of decreasing intensity of postoperative pain and increasing patient satisfaction [16]. Our study did not reveal any statistically significant difference in pain intensity during first 10 hours after surgery. However, during the night, the intensity of pain was higher in the group receiving morphine via PCA. One of the likely reasons might be that patients fall asleep and so do not press the PCA button on time. Moreover, the MI-patients required maximal additional analgesia (metamizole and acetaminophen) besides regular morphine therapy, which most probably resulted in the lower level of pain. The patients from MPCA group manifested better hemodynamic stability, which is a major advantage of this technique as compared with intermittent morphine injections. Finally, patients’ satisfaction was highly rated in both groups, which strongly suggests that morphine administration in postoperative analgesia is both desirable and justified.

For this study, we selected patients who underwent AAA surgery because it is considered one of the most painful procedures, and we thought it would be a good indicator of surgical stress. This pathology is much more common in male patients; thus, using homogenous groups prevented the influence of gender on the results.

Neuroendocrine response to stress and activation of hypothalamic-pituitary-adrenal axis result in elevated secretion of catecholamines and glucocorticoids [7] [17]. Cortisol is widely known as stress hormone. It is a catabolic hormone released from the adrenal gland cortex in response to physical and psychical stress [8] [16]. The major role of cortisol is to mobilize the body under stress conditions, mainly through its effects on energy and metabolism [18] [19] [20]. It is well established that 3–10% of total circulating cortisol is free (unbound), whereas the majority of the remaining cortisol shows high affinity of binding to cortisol-binding globulin. Albumin binds some 10–15% of cortisol with low affinity [21]. In general, cortisol level increases with the pain intensity [18]. Serum cortisol values increase within 15 minutes after acute stress, but decreases to basal levels as soon as the balance is re-established [22].

Salivary cortisol is a valuable potential marker of vulnerability to stress disorder. Biochemical presentation of hypercortisolemia, that is, increased concentration of salivary cortisol, reflects the enhanced »capacity« of the central adrenergic system. The measure ment of salivary cortisol concentration has several advantages over the conventional determination of total blood concentrations. The sampling procedure for salivary cortisol measurement is simple, non-invasive and stress-free, whereas blood sampling might be stressful and thus cause consequent increase in cortisol levels [23] [24]. Salivary cortisol correlates with the level of free serum cortisol [19] [25], which was confirmed in our study. The time lag for manifestation of changes in salivary cortisol is very short, only 1 to 2 minutes [26].

The first day post-surgery is still the period when the highest level of stress is expected, especially after major surgery such as AAA, which is due to high intensity of pain despite therapy, as well as to stress associated with hospitalization. The results of our study revealed that, on the first postoperative day in the morning, serum cortisol levels were within reference range, close to the upper reference limit. The values were almost identical in both groups. However, the values for salivary cortisol were several times higher in both groups, yet without statistically significant differences between the groups. During the night, the intensity of pain was higher in MPCA-group, which resulted in increased levels of salivary cortisol in the morning being 116.3 nmol/L as compared with 47.1 nmol/L in MI-group.

Considering the precisely and strictly defined criteria for qualifying a parameter as a biomarker, a longitudinal study which would compare the preoperative and postoperative values might offer some guidelines for salivary cortisol as a candidate for »state« or »trait« biomarker. Anyway, salivary cortisol could be considered as a marker for overall vulnerability to psychopathological manifestations occurring during perioperative period, which is in compliance with literature data [27].

Our study has several limitations. It is a single-center study, and the number of participants was relatively small. Both the pain evaluation according to NRS and the evaluation of patients’ satisfaction with analgesia are subjective methods, which might affect the results.

## Conclusion

According to our results study demonstrate that opioid analgesic morphine proved excellent for managing the acute postoperative pain after AAA repair treatment and because of that our next study planned to give surgical patients various anti-stress medicine as adjuvant therapy and examine their effects on acute postoperative pain after surgical interventions. Both analgesic techniques using morphine, with all their advantages and drawbacks, are acceptable and legitimate choice for postoperative analgesia. Our study confirmed that salivary cortisol is a more sensitive stress biomarker in surgical patients as compared to serum cortisol. Nevertheless, further studies are needed to provide additional evidence.

## Dodatak

### List of abbreviations

MI, morphine intermittently;<br>MPCA, morphine patient-controlled analgesia;<br>AAA, abdominal aortic aneurysm;<br>PCA, patient-controlled analgesia;<br>HPA, hypothalamus- pituitary-adrenal;<br>CRF, corticotrophin-releasing factor;<br>NRS, numerical rating scale;<br>CMIA, chemiluminescent microparticle immunoassay;<br>ECLIA, electro-chemiluminescence immunoassay

### Conflict of interest statement

All the authors declare that they have no conflict of interest in this work.
